# Effect of Core Versus Hip Strengthening on Knee Function in Adults With Knee Osteoarthritis: A Randomized Controlled Trial

**DOI:** 10.7759/cureus.85967

**Published:** 2025-06-13

**Authors:** Vrushali Jadhav, Shilpshree Palsule

**Affiliations:** 1 Occupational Therapy, Occupational Therapy (OT) Training School and Centre, Seth Gordhandas Sunderdas (GS) Medical College and King Edward Memorial (KEM) Hospital, Mumbai, IND

**Keywords:** core stability, functional outcome, hip strengthening, knee osteoarthritis, occupational therapy

## Abstract

Background

Osteoarthritis (OA) is a prevalent condition characterized by joint pain, functional limitation, and reduced quality of life, particularly affecting older adults, with a higher prevalence in women. OA impacts daily activities, such as walking, climbing stairs, and driving, and is one of the leading causes of pain and disability worldwide. This further affects a person's ability to carry out their activities of daily living and reduces quality of life as a consequence. Exercise remains a core recommendation for managing knee osteoarthritis (OA), and structured exercise programs have been shown to improve knee-related and overall health outcomes. Existing literature supports the effectiveness of core stability and hip strengthening exercises when added to knee strengthening for the management of OA knee. However, there is limited research comparing these two approaches. This study aims to evaluate the effect of supervised core stability versus hip strengthening exercises, alongside conventional occupational therapy, on improving functional outcomes, mobility, and strength in OA knee patients.

Methodology

Patients diagnosed with unilateral osteoarthritis of the knee, grades 1, 2, and 3 according to the Kellgren-Lawrence( KL) grading system, managed conservatively, with no history of trauma or surgery of symptomatic knee after taking approval from the Institutional Ethics Committee of Seth GS Medical College and KEM Hospital were categorized into group A (Core) and group B (Hip) using convenient sampling. A total of 34 patients, both males and females, between 40 and 60 years of age were allocated into group A or B, and were evaluated at baseline, 2 weeks, 4 weeks, and 6 weeks on the knee osteoarthritis outcome score (KOOS), 30-second chair stand test, and the modified Oxford manual muscle grading system. Both groups were given conventional occupational therapy treatment.

Results

Both the Core and Hip groups showed a statistically significant improvement in KOOS, muscle strength of knee flexors and extensors, and the number of repetitions on the 30-second chair stand test. When the Core and Hip groups were compared, the improvement in these parameters was statistically insignificant.

Conclusion

Both hip and core strengthening exercises are equally effective in improving muscle strength, pain, quality of life, and activities of daily living, leading to improvement in KOOS scores in patients with OA knee.

## Introduction

Osteoarthritis (OA) refers to a clinical syndrome of joint pain accompanied by varying degrees of functional limitation and reduced quality of life. It is the most common form of arthritis that causes disability, in which the age-specific prevalence of symptomatic OA increases with age, with women being more prevalent than men. It is one of the leading causes of pain and disability worldwide [1]. Pain, swelling, and stiffness lead to physical inactivity, which has an impact on participation in activities of daily living (ADL) and quality of life, and can be important consequences of osteoarthritis [2]. It is well-established that symptoms of OA can make it difficult for individuals to engage in ADL, such as functional mobility, driving, ascending or descending stairs, and transferring and placing objects. However, there is limited literature on the consequences of osteoarthritis on the functional performance of patients who are undergoing conservative treatment [3]. Most cases of osteoarthritis have no known cause and are referred to as primary osteoarthritis. Primary osteoarthritis can be age-related, idiopathic, and have no associated traumatic cause. It can present in different forms, such as localized, generalized, or erosive osteoarthritis. Secondary osteoarthritis is caused by an underlying disease, mechanical misalignment, or as a post-traumatic consequence. In focus groups, patients described the early stage of OA pain progression as activity-induced intermittent pain, which triggers avoidance of high-impact activities. Exercise remains a core recommendation for knee OA and may have beneficial effects on knee-related and health-related outcomes [4]. Exercise training programs improve muscle strength, and resistance training has been shown to significantly increase skeletal muscle mass and strength associated with an increase in physical activities, ultimately improving health outcomes and ADL of people diagnosed with osteoarthritis of the knee. According to the 2019 Osteoarthritis Research Society International (OARSI) Guidelines for the non-surgical management of knee osteoarthritis, which are comprehensive, patient-centered, and facilitate individualized treatment decisions, structured land-based exercise programs, weight management along with exercises, and alternative therapies, such as Tai Chi and Yoga, were recommended for all patients with knee OA, irrespective of the type of OA. Education and self-care techniques related to OA can improve quality of life and are considered a standard-of-care practice by the panel [5]. The rationale of the study was that there is abundant literature that proves the efficacy of core stability or hip strengthening when added to knee strengthening for osteoarthritis of the knee. Studies comparing the effects of the two are few, which implies that there needs to be further studies that show whether hip strengthening or core stability exercises, along with knee strengthening, have a better outcome in osteoarthritis of the knee [6-8]. The aim of the study was to determine the effect of supervised core stability versus hip strengthening exercises along with a conventional therapy program in patients with osteoarthritis of the knee. The primary objectives of the study were to study the effect of core stability vs. hip strengthening, along with knee strengthening, on functional outcomes pre- and post-intervention using KOOS in patients with osteoarthritis of the knee. The secondary objectives of the study were to study the effect of core vs. hip strengthening on functional mobility and strength using the 30-sec chair stand test and to compare strength in both groups using manual muscle testing by the modified Oxford system.

## Materials and methods

A randomized controlled trial was conducted at the Occupational Therapy Department of KEM Hospital. Subjects included in the study were males (N=26) and females (N=42) between 40 and 60 years of age, diagnosed with primary unilateral osteoarthritis of the knee, who had clinical and radiographic symptoms of osteoarthritis of the knee grades 1, 2, and 3 according to the Kellgren-Lawrence Classification of Osteoarthritis, and were managed conservatively. Subjects were excluded if they had any major trauma or surgery to the symptomatic knee or lower limb, neuromuscular disorders, rheumatoid arthritis, gout, history of stroke and cardiovascular disease, ligament injuries and lower extremity fractures, intra-articular corticosteroid injections within six months (current) or oral corticosteroid use within four weeks (past), or any diagnosed spinal pathology. Subjects were recruited using convenience sampling, and patients were randomized into two groups using a table of random numbers. The sample size was determined using the estimates of mean and standard deviation values from Guliya et al. [9], keeping 80% power and the type 1 error as 5%, 34 subjects per group (total 68 subjects) completed the trial at the endpoint of follow-up. After obtaining approval from the Institutional Ethics Committee of Seth GS Medical College and KEM Hospital (IEC number: EC/58/2022). The study was registered with the Clinical Trials Registry, India (Registration No: CTRI/2023/02/049448, Reference No: REF/2022/12/061845). Eligible candidates were randomized to either group A (Core) or group B (Hip). Patients in both groups were evaluated on outcome measures such as the Knee Injury and Osteoarthritis Outcome Score (KOOS) [10], the 30-second chair stand test, at baseline, two weeks, four weeks, and six weeks. Group A was given core stability exercises + conventional occupational therapy treatment for osteoarthritis of the knee. Group B was given hip strengthening exercises + conventional occupational therapy treatment for osteoarthritis of the knee. Three sets of 10 repetitions each were performed for the muscle groups exercised. The case record form had demographic data, the chief complaints, a knee joint examination, which included tenderness, swelling, pain, range of motion (ROM), and grading of the stage of OA. The muscle strength of the hip and knee was assessed by manual muscle testing.

**Table 1:** Conventional occupational therapy treatment for osteoarthritis knee Adapted from: Carolyn Kisner (2017) [11], Brotzman SB (2011) [12], Pendleton HM (2018) [13]

Conventional Occupational Therapy Treatment
Patient Education
Joint Protection Techniques and Fatigue Management
Advice on Activities of Daily Living
Stationary Cycling
Passive, Active, Assistive, or Active ROM
Multiple Angle Isometrics for the Quadriceps and Hamstrings
Isotonic Strengthening of Knee Flexors and Extensor Muscles Using Progressive Resistive Exercises and Enabling Activities
Use of an Assistive Device for Ambulation if Necessary
Ambulation Training – Practice Walking on a Variety of Terrains and Inclines, Including Changes of Direction, First With Assistance, Then Independently
Functional Adaptations, Such As Advice on the Use of a Commode for Toilet Purposes
Functional Training - Climbing Steps, Sitting Down, Rising From Chairs and Commodes, Using Safe Body Mechanics to Lift Objects From the Floor
Balance Activities for Static and Dynamic Balance

**Table 2:** Group A: core stability exercises Adapted from: Carolyn Kisner (2017) [11], Akuthota V (2008) [14], Kutty N (2021) [15], Wisnubrata M (2020) [16], Hernandez D (2019) [17]

0-2 weeks	2-4 weeks	4-6 weeks
Static abdominals	Quadruped static abdominals	Quadruped Static abdominals
Bridging	Bridging with unilateral straight leg raise	Bridging with marching.
Abdominal curls with the arm outstretched	Abdo curls with arm behind the head	Abdo curls with arms across the chest.
Sitting on the therapy ball	Sitting on a therapy ball and transferring objects with feet on the floor	Sitting on a therapy ball, transferring objects with feet off the ground

**Table 3:** Group B: hip strengthening exercises Adapted from: Carolyn Kisner (2017) [11], Lun V (2015) [18], Dabholkar T (2016) [19]

0-2 weeks	2-4 weeks	4-6 weeks
Static Gluteus	Standing Hip extension with a 0.5-kg weight cuff on the ankle	Standing hip extension using a 1-kg weight cuff on the ankle
Clam Shell Exercise	Clamshell with mild resistance TheraBand	Clamshell with moderate resistance TheraBand
Wall Squats	Sit to stand with 90-degree hip flexion	Sit to stand on a chair with a weight in the hand
Step Ups	Step up with increasing height	Step up with a weight in the hand

## Results

Statistical procedures

The data collected were compiled using IBM SPSS Statistics Version 26.0. Intra-group comparisons were done using Friedman’s test at baseline, two weeks, four weeks, and six weeks. The mean and standard deviation of numerical data in each group were depicted and compared using the chi-square test. An inter-group comparison was done using the Mann-Whitney U test at two weeks, four weeks, and six weeks (Tables 4, 5). p<0.05 was considered statistically significant.

**Table 4:** Comparison of muscle strength between group A and group B The Mann-Whitney U test was conducted to compare muscle strength using manual muscle testing between two groups, Hip and Core, at two weeks, four weeks, and six weeks. As per Table 4, the results revealed that there is a significant difference in knee flexors and extensors in both the Hip and Core groups at four weeks compared to baseline and at six weeks compared to baseline. The Core group had a significantly higher sum of ranks compared to the Hip group.

Scale	Group	P value
2weeks_Baseline Flexors	Hip	0.11
	Core	
4weeks_Baseline Flexors	Hip	0.026*
	Core	
6weeks_Baseline Flexors	Hip	0.002*
	Core	
4weeks_2weeks Flexors	Hip	0.707
	Core	
6weeks_2weeks Flexors	Hip	0.076
	Core	
6weeks_4weeks Flexors	Hip	0.096
	Core	
2weeks_Baseline Extensor	Hip	0.268
	Core	
4weeks_Baseline Extensor	Hip	0.01*
	Core	
6weeks_Baseline Extensor	Hip	0.001*
	Core	
4weeks_2weeks Extensor	Hip	0.223
	Core	
6weeks_2weeks Extensor	Hip	0.023*
	Core	
6weeks_4weeks Extensor	Hip	0.154
	Core	

**Table 5:** Comparison of the number of repetitions on the chair stand test between Group A and Group B The Mann-Whitney U test was conducted to compare the number of repetitions on the 30-second chair stand test between two groups, Hip and Core, at two weeks, four weeks, and six weeks. As per Table 5, the results revealed that there is a significant difference between the Hip and Core groups at four weeks compared to two weeks. The Core group was observed to have a higher sum of ranks compared to the Hip group.

Scale	Group	P value
2weeks_Baseline No of Rep	Hip	0.568
	Core	
4weeks_Baseline No of Rep	Hip	0.533
	Core	
6weeks_Baseline No of Rep	Hip	0.958
	Core	
4weeks_2weeks No of Rep	Hip	0.02*
	Core	
6weeks_2weeks No of Rep	Hip	0.776
	Core	
6weeks_4weeks No of Rep	Hip	0.128
	Core	

Intra-group analysis

**Figure 1:**
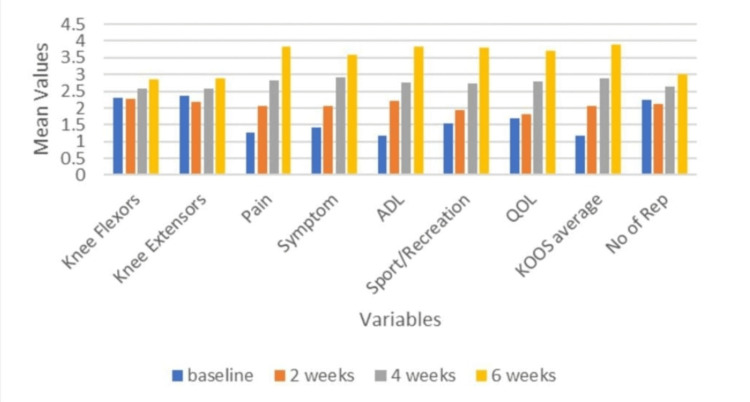
Intra-group analysis of group A (Core) The Friedman test results reveal statistical significance at baseline, two weeks, four weeks, and six weeks for knee flexors, knee extensors, and number of repetitions (p < 0.05). However, pairwise comparison using Bonferroni correction for the variables Knee Flexors and Knee Extensors did not identify specific time points where these differences reached statistical significance. The Friedman test results are statistically significant at baseline, two weeks, four weeks, and six weeks for components of the knee injury osteoarthritis outcome score (KOOS) scale that is pain, symptom, activity of daily living (ADL), sport/recreation, quality of life (QOL), KOOS average (p < 0.05).

**Figure 2:**
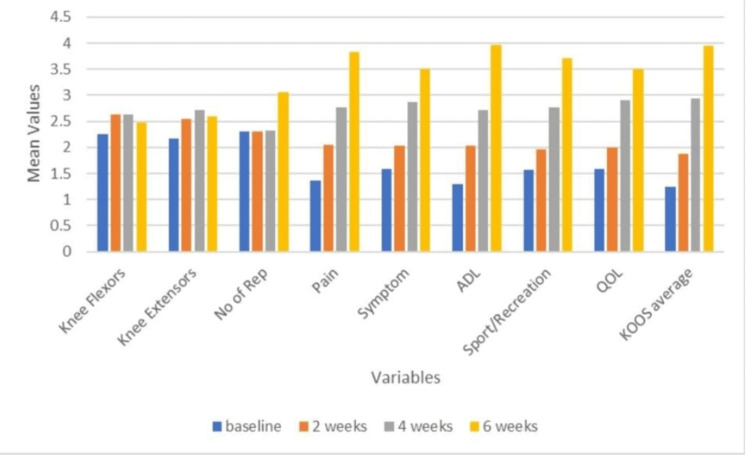
Intra-group analysis of group B (Hip) The Friedman test results reveal statistical significance at baseline, two weeks, four weeks, and six weeks for knee flexors, knee extensors, and number of repetitions (p < 0.05). However, pairwise comparison using Bonferroni correction for the variables knee flexors and knee extensors did not identify specific time points where these differences reached statistical significance. The Friedman test results reveal statistically significant at baseline, two weeks, four weeks, and six weeks for components of the knee injury osteoarthritis outcome score (KOOS) scale that is pain, symptom, activity of daily living (ADL), sport/recreation, quality of life (QOL), KOOS average (p < 0.05).

Figures 1, 2 show the intra-group analyses of groups A and B.

Inter-group analysis 

The Mann-Whitney U test was conducted to compare KOOS scale parameters between two groups, Hip and Core, at baseline, two weeks, four weeks, and six weeks. None of the variables were found to be statistically significant.

**Figure 3:**
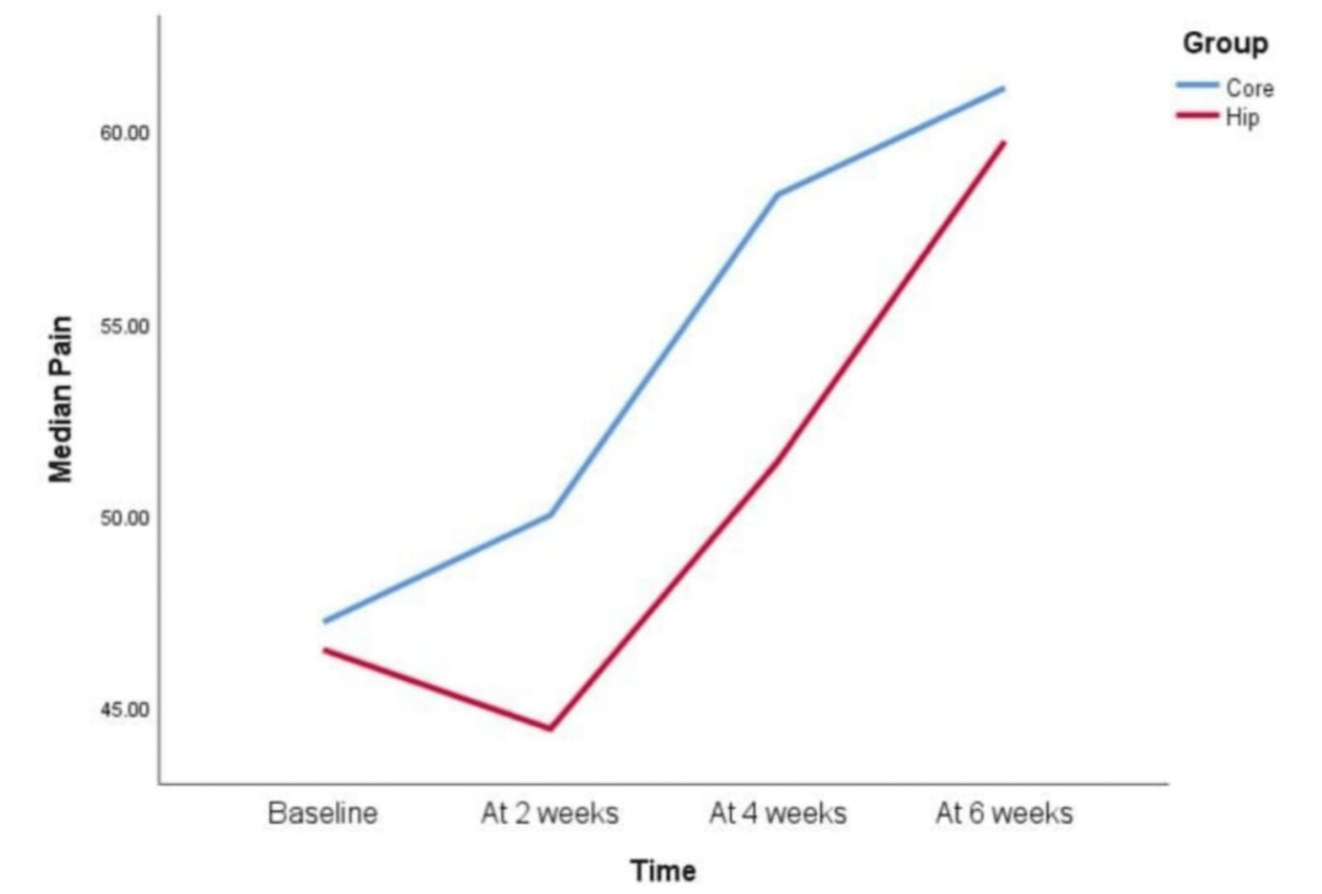
KOOS scale pain component at baseline, two weeks, four weeks, and six weeks Both the Hip and Core groups performed similarly with respect to the pain component of the knee injury and osteoarthritis outcome score (KOOS); that is, 0-2 weeks and 2-4 weeks did not show statistically significant improvement. From the fourth week onward, the pain score improved in both groups, which is evident from the fact that there was statistically significant improvement when pain scores were compared between 4-6 weeks and 2-6 weeks. Both groups showed a statistically significant improvement when scores of baselines were compared with the sixth week score. The improvement in scores when compared in 0-2 weeks and 2-4 weeks was not statistically significant.

**Figure 4:**
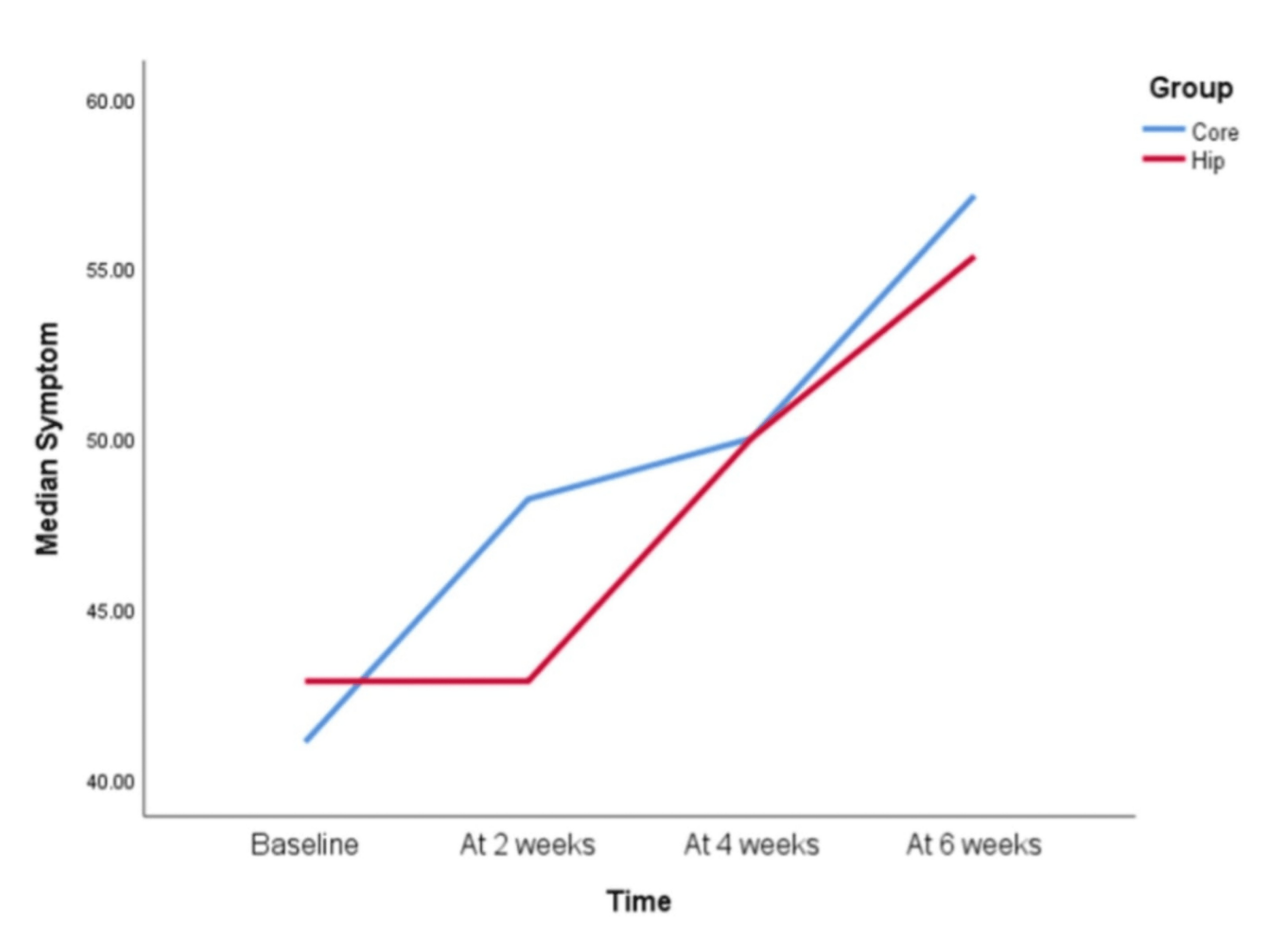
KOOS scale symptom component at baseline, two weeks, four weeks, and six weeks Both the Hip and Core groups performed similarly. In 0-2 weeks and 4-6 weeks, there was no statistically significant difference in improvement in both groups on the knee injury and osteoarthritis outcome score (KOOS). In all other timelines, that is, 2-4 weeks and 2-6 weeks, there was a statistically significant improvement in scores. The scores at 0-4 weeks and 0-6 weeks also showed a significant difference in both groups.

**Figure 5:**
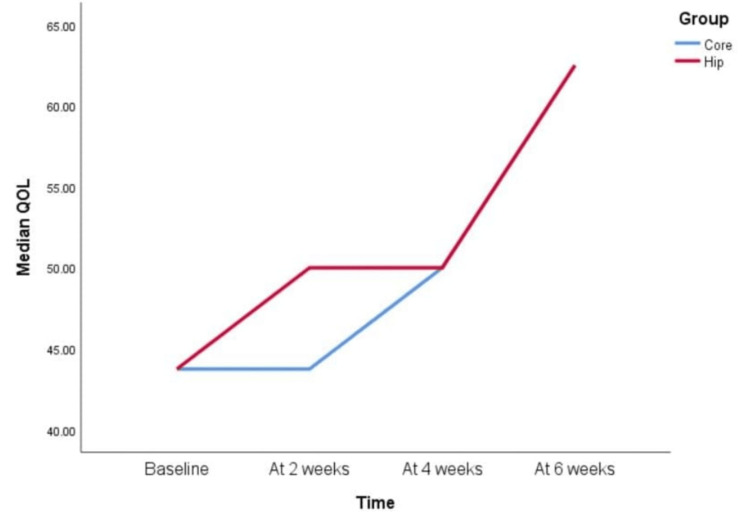
KOOS scale QOL component at baseline, two weeks, four weeks, and six weeks On the knee injury and osteoarthritis outcome score (KOOS) scale, quality-of-life (QOL) scores of both groups did not show improvement when compared at 0-2 weeks. However, when baseline scores were compared to four weeks and six weeks, there was a statistically significant difference observed. Both groups showed statistical differences in scores at 2-4 weeks, but only the core group showed an improvement at 4-6 weeks. This means that the Hip group remained static. When scores of both groups were compared for 2-6 weeks, there was a statistically significant difference observed.

**Figure 6:**
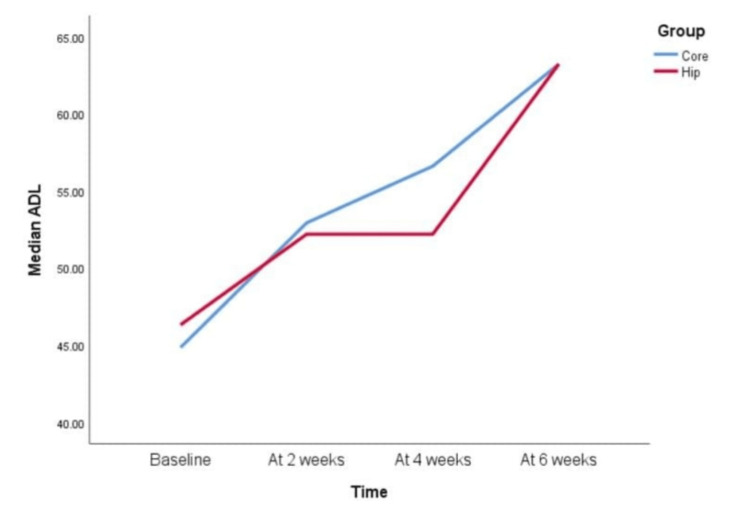
KOOS scale ADL component at baseline, two weeks, four weeks, and six weeks In the knee injury and osteoarthritis outcome score (KOOS) scale, activities of daily living (ADL) component scores, only the Core group showed significant improvement in scores in the first 2 weeks, i.e., 0-2 weeks. Both groups showed statistically insignificant differences when scores were compared at 2-4 weeks. Both groups showed significant improvement when scores were compared at 4-6 weeks and 2-6 weeks. Both the groups’ scores, when compared from 0-4 weeks and 0-6 weeks, there was a statistically significant difference observed.

**Figure 7:**
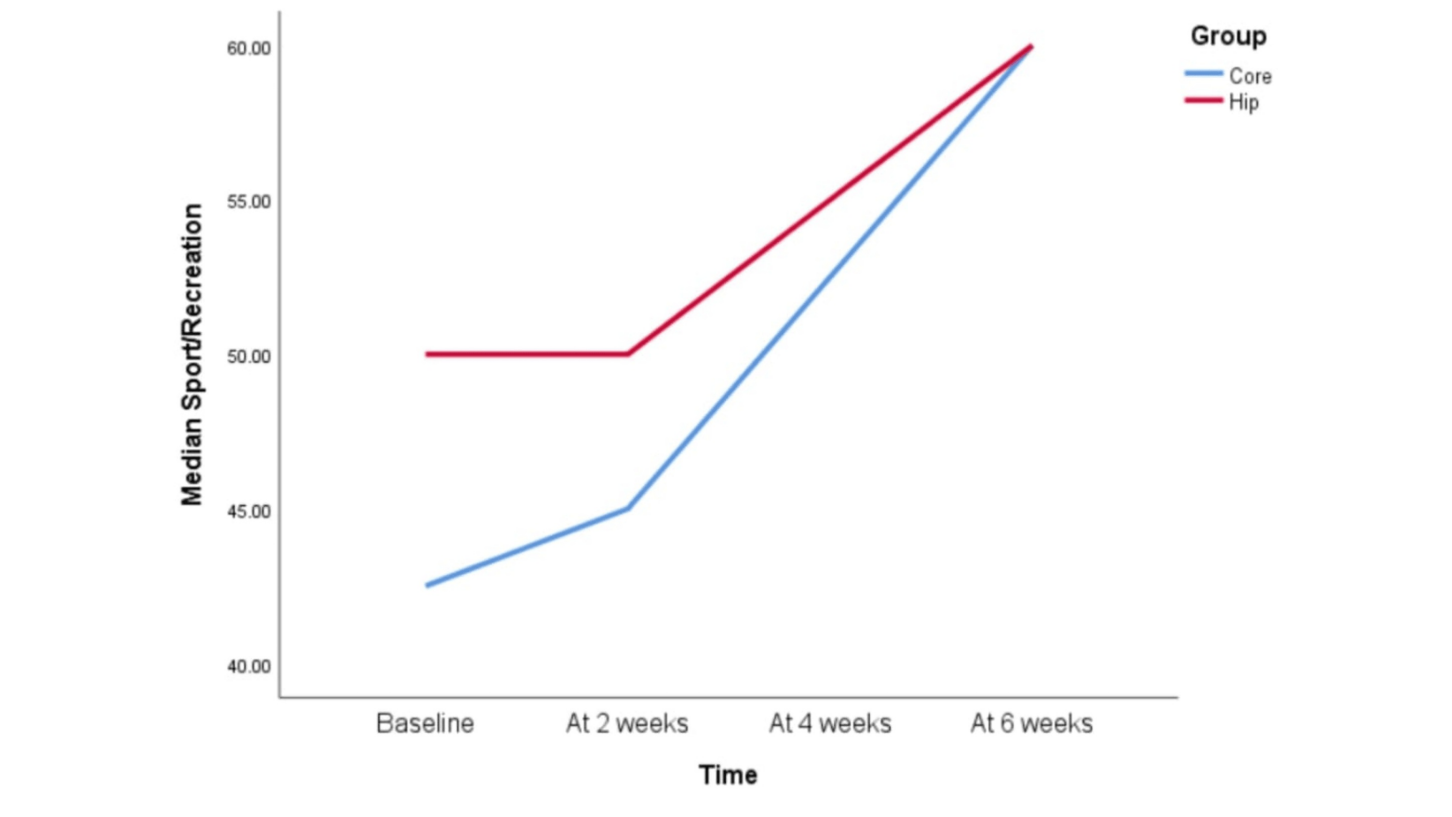
KOOS scale sports/recreation component at baseline, two weeks, four weeks, and six weeks Both the Hip and Core groups performed similarly with respect to the sports and recreation component of the knee injury and osteoarthritis outcome score (KOOS), that is, 0-2 weeks and 2-4 weeks did not show statistically significant improvement. From the fourth week onward, sports and recreation scores improved in both groups, which is evident from the fact that there was statistically significant improvement when scores were compared between 4-6 weeks and 2-6 weeks. Both groups showed statistically significant improvement when scores of baselines were compared with the sixth week score, i.e., 0-6 weeks. The improvement in scores when compared in 0-2 weeks and 2-4 weeks was not statistically significant.

**Figure 8:**
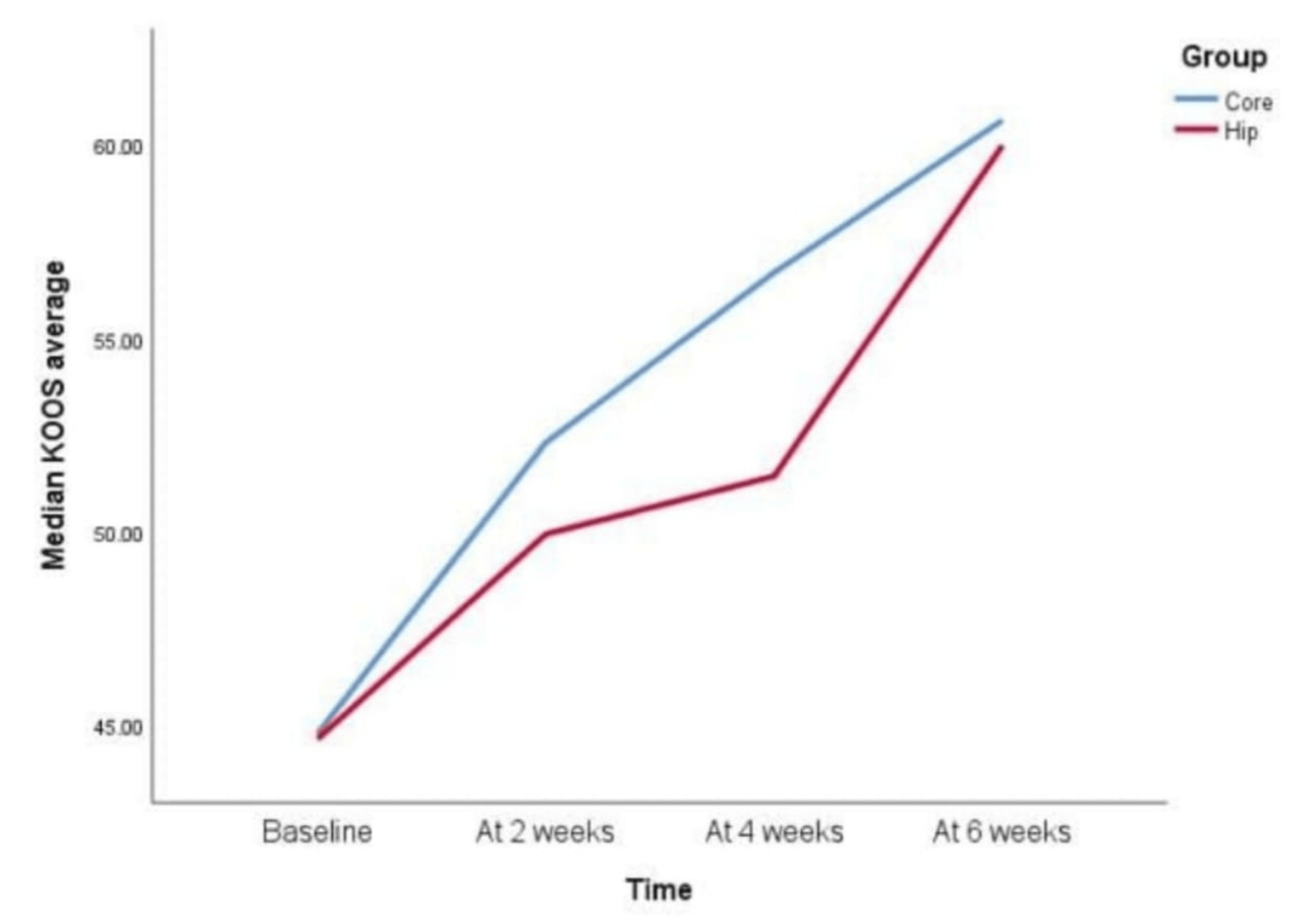
KOOS scale KOOS average component at baseline, two weeks, four weeks, and six weeks In the knee injury and osteoarthritis outcome score (KOOS), the KOOS average component, a 0-2 weeks average score improvement was seen in the Core group but not in the Hip group. Later, there was improvement in the 2-4 weeks in the Hip group but not in the Core group. This means that the Core group showed improvement at 0-2 weeks, but later, it remained static or slow. At 4-6 weeks, both groups were comparable, with similar results in both groups. Also both groups showed a statistically significant difference when scores were compared from baseline to the fourth week and baseline to the sixth weeks; that is, 0-4 weeks and 0-6 weeks respectively.

However, when KOOS scale parameters were compared between the Hip group and the Core group, the following pattern was observed for each component (Figures 3-8).

## Discussion

In our six-week intervention study, we observed that both the Hip and Core groups showed similar improvements in the pain component of the KOOS scale. Pain scores improved from the fourth to the sixth week in both groups compared to baseline. A literature review by Wisnubrata and Zharfan indicated that core muscle activation provides proximal stability to the knee, reducing the load on the knee joint, thus alleviating pain in OA knee patients [16]. This finding aligns with Hernandez’s study, which found reduced pain and perceived disability in the core group due to improved knee joint biomechanics [17]. Lun et al., in their study on hip strengthening for OA knee patients, suggested that pain score improvements in the KOOS scale post-intervention could be attributed to increased pelvic stability and improved dynamic lower extremity alignment, which helps reduce the knee adduction moment [18,19]. The OARSI 2014 guidelines recommend strength training exercises to alleviate pain and enhance physical function. Several studies have also shown that exercise therapy relieves pain and reduces disability, potentially due to decreased activity in pain fibers, the effect of motor neurons, or endorphin release from the CNS post-exercise. These benefits were observed in our study as well [6]. While the guidelines suggest at least 12 supervised sessions of exercise therapy, our study limited patient follow-ups to feasibility concerns, emphasizing a home exercise program instead.

In our study, the KOOS QOL component showed statistically significant improvement from the second to the fourth weeks in the Hip group and from the second to the sixth week in the Core group. In an RCT, adjuvant hip abductor exercises combined with quadriceps exercises improved QOL after two weeks compared to the control group, which showed improvement only after four weeks [20]. They suggested this might be due to the activation of the gluteus medius. Our study found similar results for the Hip group, showing improvement from the second week, but the scores plateaued after four weeks, whereas the RCT reported continued improvement up to the tenth week in their study. While no similar literature exists for the core group, the improvement in QOL could likely be due to better mobility, as evidenced by improvements in the ADL scores. Regarding the ADL component of the KOOS scale, the hip group showed improvement only from the fourth to the sixth weeks, while the core group showed significant improvement from the second to the fourth weeks, followed by further improvement from the fourth to the sixth weeks. A significant difference was observed in both groups’ scores when comparing the baseline to the fourth week and the baseline to the sixth week. Yuenyongviwat et al. found that the addition of hip strengthening exercises improved ADL scores more quickly than quadriceps exercises alone, but with a small effect size [20]. Singh S found significant improvements in Western Ontario and McMaster Universities Osteoarthritis Index (WOMAC) scores after a six-week hip strengthening intervention for OA knee patients [21]. Daste et al. suggested that weak core muscles result in poor endurance, leading to increased knee joint load during dynamic movements, which can worsen symptoms in OA patients [22]. The core muscles help reduce stress on the knee joint by providing proximal stability, improving coordination, and promoting smoother, more secure lower extremity movement, thereby enhancing ADL performance.

The KOOS average score in our study showed significant improvement at two, four, and six weeks in the core group, consistent with a study by Flowers et al., which found significant improvements in overall KOOS scores due to biomechanical changes such as reduced knee adduction moments, improved gait speed, and better functional ability [23]. Core stabilization training, which focuses on lumbar and abdominal muscles, has been shown to improve balance and mobility, with better coordination between trunk and extremity movements. Granacher et al. found that core stability training has a more pronounced effect on balance and mobility in older adults compared to traditional strength training [24]. These improvements in pain, QOL, and ADL scores in both the hip and core groups likely contributed to the overall improvement in KOOS scores.

At the sixth week, both the Hip and Core groups showed statistically significant improvements in knee flexor and extensor strength. These findings align with the Ottawa Panel guidelines, which support strength training for reducing pain and improving stability [25]. In our study, both the core and hip groups showed significant improvements in the number of repetitions in the 30-second chair stand test at six weeks compared to baseline, with the core group showing more significant improvement between the second and sixth weeks. However, the timeline of improvement in the Hip group was less clear. Factors such as learning, motivation, pain, and fatigue may have influenced performance on the 30-second chair stand test, as suggested by Gill et al. [26]. Pain, in particular, is known to significantly impact performance in individuals with symptomatic knee OA. The improvement in repetitions likely reflects reduced pain, as indicated by the pain component of the KOOS. When compared to normative data from the Indian population, our study participants had lower scores on the CST, suggesting that the six-week program may not have been sufficient to achieve normal functional levels, as compared to healthy adults of the same age group. This suggests that exercise therapy may need to be continued beyond the six-week protocol to fully restore normal function [27-30].

The limitations of this study include the frequency of follow-up, which may have impacted the consistency of data collection. Additionally, the small sample size limits the generalizability of the findings. There was an unequal representation of males and females, which could introduce gender-related biases. Important factors, such as BMI and occupations, were not considered during the inclusion process, potentially affecting the study's outcomes. Lastly, the severity of knee OA was not categorized, which may have influenced the interpretation of results.

## Conclusions

Both Core and Hip strengthening exercises, along with knee strengthening exercises, are equally effective in osteoarthritis of the knee for 40-60 years. Individual components of the KOOS scale - pain, sports and recreation, KOOS average, ADL, QOL, and 30-second chair stand test values - improved from baseline to six weeks in both groups. Knee strengthening exercises performed as per the standard of care for osteoarthritis of the knee improved both quadriceps and hamstring strength in both study groups, and this effect is more evident after four weeks of therapy.
